# Genome-Wide Identification and Characterization of the Calmodulin-Binding Transcription Activator (CAMTA) Gene Family in Plants and the Expression Pattern Analysis of *CAMTA3/SR1* in Tomato under Abiotic Stress

**DOI:** 10.3390/ijms23116264

**Published:** 2022-06-03

**Authors:** Hua Fang, Peng Wang, Fujin Ye, Jing Li, Meiling Zhang, Chunlei Wang, Weibiao Liao

**Affiliations:** 1 College of Horticulture, Gansu Agricultural University, Lanzhou 730070, China; fanghua1610@163.com (H.F.); wangp@st.gsau.edu.cn (P.W.); yfj18894546481@163.com (F.Y.); lj19990716@163.com (J.L.); wangchunlei@gsau.edu.cn (C.W.); 2 College of Science, Gansu Agricultural University, Lanzhou 730070, China; zhangml@gsau.edu.cn

**Keywords:** tomato, phylogenetic relationships, evolutionary relationships, conservative structural domain, protein clustering interaction, tissue-specific expression, abiotic stresses

## Abstract

Calmodulin-binding transcription activator (CAMTA) plays an important regulatory role in plant growth, development, and stress response. This study identified the phylogenetic relationships of the CAMTA family in 42 plant species using a genome-wide search approach. Subsequently, the evolutionary relationships, gene structures, and conservative structural domain of *CAMTA3/SR1* in different plants were analyzed. Meanwhile, in the promoter region, the cis-acting elements, protein clustering interaction, and tissue-specific expression of CAMTA3/SR1 in tomato were identified. The results show that *SlCAMTA3/SR1* genes possess numerous cis-acting elements related to hormones, light response, and stress in the promoter regions. SlCAMTA3 might act together with other Ca^2+^ signaling components to regulate Ca^2+^-related biological processes. Then, the expression pattern of *SlCAMTA3/SR1* was also investigated by quantitative real-time PCR (qRT-PCR) analysis. The results show that *SlCAMTA3/SR1* might respond positively to various abiotic stresses, especially Cd stress. The expression of *SlCAMTA3/SR1* was scarcely detected in tomato leaf at the seedling and flowering stages, whereas *SlCAMTA3/SR1* was highly expressed in the root at the seedling stage. In addition, *SlCAMTA3/SR1* had the highest expression levels in flowers at the reproductive stage. Here, we provide a basic reference for further studies about the functions of *CAMTA3/SR1* proteins in plants.

## 1. Introduction

Divalent cation calcium (Ca^2+^) ions are crucial intracellular second messengers that play a dominant role in many biological processes and signal transduction pathways. Mitochondria control the uptake and release of Ca^2+^ to ensure its homeostasis in the cell by regulating cellular Ca^2+^ levels. Thus, at the mitochondrial level, Ca^2+^ has a dual role, being involved in important physiological processes such as Adenosine triphosphate (ATP) production and regulation of mitochondrial metabolism and in pathophysiological processes such as cell death, cancer progression, and metastasis [1]. Calmodulin (CaM) senses and interprets transient increases in intracellular [Ca^2+^] cyt levels, ultimately leading to conformational changes and the activation of CaM [2]. Multiple cellular processes, including stress responses and plant development, are known to be regulated in plants by CaM [3]. Transcription factors (TFs) play a pivotal role in long-term plant stress resistance responses by integrating upstream environmental inputs to regulate the expression of several downstream genes, thereby coordinating different physiological processes including chlorophyll content and photosynthesis levels maintenance.

CAMTA (calmodulin-binding transcription activator), as one of the transcription factors, has been involved in performing important functions in modulating plant stress responses and development in a Ca^2+^/CaM-driven modus [4,5,6,7]. Signal-responsive (SR) /CAMTAs were first discovered while screening for CaM-binding proteins in *Nicotiana tabacum* and have since been found in plants and animals. Thus far, six CAMTA genes have been reported in *Arabidopsis thaliana* [4,8,9,10]. With the advent of genome sequencing, the identification of similar CAMTAs has been performed in other plant species, including *Oryza sativa* L. [11], *Solanum lycopersicum* [12], *Vitis vinifera* [13], *Glycine max* [14], *Medicago truncatula* [15], *Zea mays* L. [16], *Fragaria ananassa* [17], *Nicotiana tabacum* [18], *Gossypium* [19], *Phaseolus vulgaris* [20,21], *Sorghum bicolor* [20,22], and so on. To date, the protein domain and gene structure of CAMTAs have been reported in detail in *Arabidopsis thaliana* [4], *Oryza sativa* L. [11], *Solanum lycopersicum* [12], *Vitis vinifera* [13], *Glycine max* [14], *Medicago truncatula* [15], *Zea mays* L. [16], *Gossypium raimondii* [19], *Nicotiana tabacum* [18], and *Phaseolus vulgaris* L. [23].

In plants, SR/CAMTAs play various roles according to different developmental signals and various environmental signals. The family of Ca^2+^/CaM-regulated SR/CAMTA transcription factors has played an important role in plant responses to postharvest biology [24] and abiotic and biotic stresses [25]. In particular, the important role of SR1 (also known as CAMTA3) in plant growth, development, and resistance to biotic and abiotic stresses has been explored. Yuan et al. found that in *atsr1* mutants, the expression of growth factor response factor 18 (ARF18) and DWARF4 (DWF4) was positively correlated with growth hormone and oleoresin sterol (BR)–mediated plant growth [26]. This suggests that AtCAMTA3/AtSR1 may be involved in plant growth and development through growth hormone and BR-mediated signaling pathways. Moreover, CAMTA3 also regulates EIN3 (ethylene insensitive 3), which contributes to ethylene-induced senescence [27]. In addition, AtCAMTA3 was found to play an important role in the resistance to *Sclerotinia sclerotiorum* invasion [28]. EMSA and CHIP analyses indicate that AtSR1 maintains a strong balance between plant immunity and growth by interacting with CGCG motifs in the upstream sequences of its potential target genes [26]. In *S. lycopersicum*, SlSR1, and SlSR3L negatively regulate plant defenses against *Botrytis cinerea* and *Pseudomonas syringae* pv. *tomato* (Pst) DC3000. In contrast, SlSR1L is involved in the positive regulation of drought stress response [29]. Under low-temperature conditions, AtCAMTA3 can also enhance cold resistance in rabidopsis by binding to conserved motif 2 (CM2) and positively regulate the expression of C-repeat binding factor 2 (CBF2: cold-inducible gene) [30]. The physiological functions of CAMTA in plants confirm its possible involvement in stress response and development.

Tomatoes have a high standing in vegetable production since they are a widely cultivated vegetable crop worldwide. As mentioned above, CAMTAs are involved in plant growth and development and stress resistance. The CAMTA gene family might play an equally important role in tomato. However, our knowledge about *SlCAMTA3/SR1* as a transcription factor in regulating the trade-offs between plant growth and abiotic stresses is limited. Studies on *CAMTA3/SR1* in tomato growth and abiotic stress responses are relatively few. It was necessary for us to elucidate the multiple functions of the CAMTA family and the mechanism of *SlCAMTA3/SR1* gene expansion in tomatoes. We suspect that the *CAMTA3/SR1* gene responds to hormones, environmental stresses, and growth and development processes in tomato. Therefore, we performed a genome-wide analysis of tomato (*Solanum lycopersicum*) and focused on functional analyses of *SlCAMTA3/SR1* transcription factors in response to abiotic stresses based on published tomato genomic information. This study will provide valuable information for further functional studies of the *SlCAMTA3/SR1* gene.

## 2. Results and Discussion

### 2.1. Identification of CAMTA Gene Family Members in Plants

**Figure 1:**
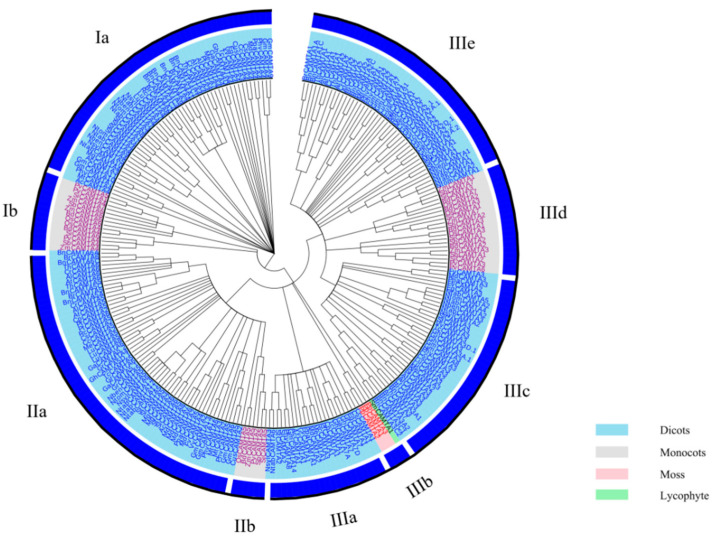
Phylogenetic relationships of 262 CAMTAs from 42 plant species. Different colored labels represent different species (sky blue, dicots; light-gray, monocots; pink, moss; light-green, lycophytes).

To better understand the evolutionary origin of the plant CAMTA proteins, a maximum-likelihood (ML) phylogenetic tree based on the alignment of full-length CAMTA proteins was constructed (Figure 1; Table S1). In all, 262 CAMTA proteins from 42 plant species were clustered into three major groups (I–III). The three groups were further divided into different subgroups. Groups I, II, and III have two, three, and five subgroups, respectively. Rahman et al. also constructed a phylogenetic tree with 200 CAMTA proteins from 35 plant species, which clustered these proteins into three groups (I–III) [20]. However, they divided groups I and II into two subgroups, while group III was separated into five subgroups [20]. A phylogenetic tree containing five crops and arabidopsis was constructed by Shen et al., and these CAMTA proteins were grouped into six groups [31]. Recently, to explore the evolutionary relationships of the CAMTA family in gossypium species, a phylogenetic tree of 17 species with 122 CAMTAs was built [19]. The CAMTA proteins were divided into five major groups (I–V), and groups I, IV, and V were further divided into two subgroups. Interestingly, all four CAMTAs from the lower non-flowering land plants (moss and lycophyte) were gathered into group IIIb, while the other 258 CAMTAs from the higher flowering land plants were distributed in the other groups. These are also in agreement with the previous study, where the lower plants (moss and lycophyte) were settled in the basal group [18,19,20]. Meanwhile, CAMTA proteins in monocots and dicots were concurrent in three major groups (I–III), which were divided clearly into different subgroups, suggesting their evolutionary divergence before the common ancestor of monocots and dicots. Here again, previous studies have shown that, before the divergence between monocots and dicots and probably even before the divergence between higher flowering plants and lower non-flowering land plants, all CAMTA proteins shared a common ancestry [19,20,22].

According to the analysis of the phylogenetic relationships, CAMTAs are involved in growth and development and stress response in both monocots and dicots, and CAMTAs are highly conserved in the evolutionary process. Despite recent advances in the identification and phylogenetics of CAMTAs, there is still much to explore in this TF family. Thus, further investigation of CAMTA proteins’ identification and phylogenetics is needed, opening new perspectives for studying the plant CAMTA family. Subsequently, CAMTA3 was selected as the target for the follow-up analysis.

### 2.2. CAMTA3/SR1 Structure in Plants

**Figure 2:**
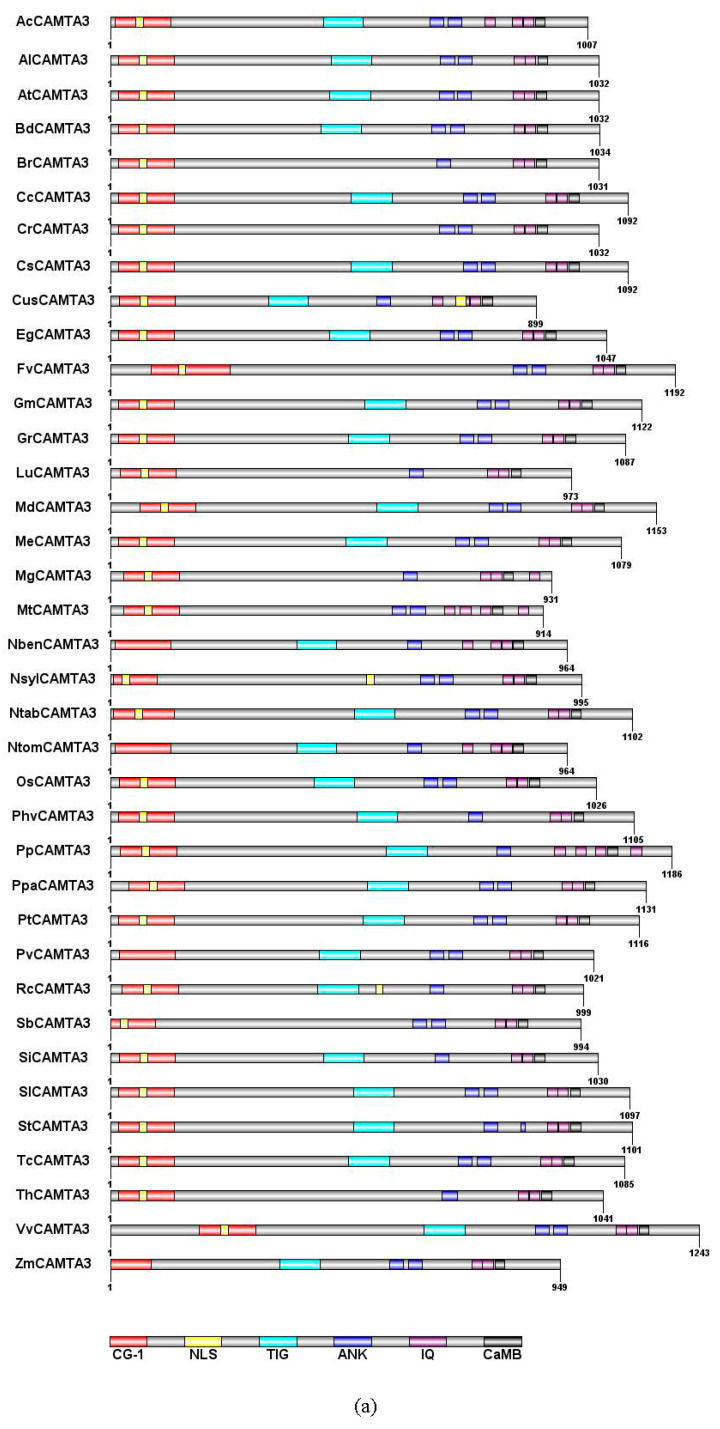

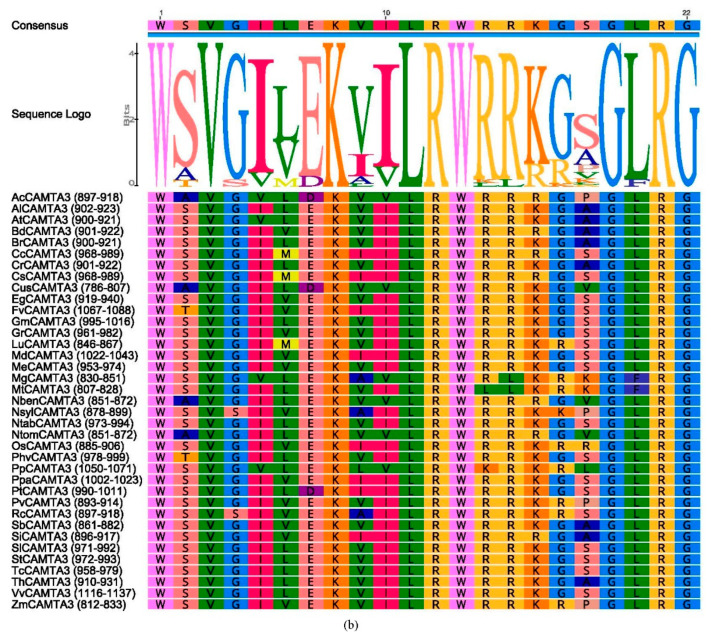
Protein conserved domain composition of CAMTA3/SR1. (**a**) Diagram of all the domains in the different plants. (**b**) Alignment of conserved CaMB domain of CAMTA3 in the different plants. The full name of the abbreviation: CG-1, sequence-specific DNA-binding domain; NLS, nuclear localization signal; TIG, transcription-associated immunoglobulin-like domain; ANK, ankyrin repeat domain; IQ, isoleucine glutamine motif; CaMBD, calmodulin-binding domain. The total height of the stack represents the information content of the relative amino acid in the position of each letter in the motif in bits. The height of the individual letter in a stack is calculated by the probability of the letter at that position times the total information content of the stack. The *X* and *Y* axes represent the width and the bits of each letter, respectively.

CAMTA family proteins, whose amino acid sequence is evolutionarily conserved in plants, contain a CG-1 DNA-binding domain, a TIG domain, ankyrin repeats (ANK), IQ motifs, and a calmodulin-binding domain (CaMB) [4,11,14,20,32]. The CG-1 structure is a domain that has about 130 amino acid residues. CAMTA3 could bind to the DNA consensus motif (G/A/C) CGCG (T/G/C) and CGTG through the CG-1 domain in *Arabidopsis thaliana* [10,33]. A recent study has revealed that 59% of CAMTA3-regulated genes contained VCGCGB and MCGTGT motifs, which are directly bound to CG-1 of CAMTA3 [34]. All the CAMTA3 from 37 species possessed the CG-1 domain, but the localization of CG-1 in CAMTA3 differed in 37 species (Figure 2a). The CG-1 domain of FvCAMTA3 and VvCAMTA3 was farther from the N-terminal than other plant CAMTA3 proteins (Figure 2a). However, CG-1 domain was reported to be missing in the rice (Os03g27080) CAMTA family [11]. Most of the CAMTA proteins were predicted to contain one nuclear localization signal (NLS) in the N-terminal. However, previous studies revealed that CAMTAs had an additional NLS in rice, located in the C-terminal [4,11,33]. According to the prediction, there were two NLSs in CusCAMTA3 and NslyCAMTA3 (Figure 2a). However, there were no NLSs in NbenCAMTA3, NtomCAMTA3, PvCAMTA3, and ZmCAMTA3, and a similar result has been reported in GmCAMTA15 [14]. TIG is a domain that participates in protein dimerization through nonspecific contact with DNA [35]. By comparing with the genetic structure and domain of 200 CAMTAs, it was found that the TIG structure first appeared in land plants [20]. At the same time, they found that, among the 200 CAMTAs, a quarter did not contain the TIG domain. We found that 24.3% of CAMTA3s belonged to the non-TIG class of 37 plant species (Figure 2a). The reason for the emergence of non-TIG CAMTA3 emerged is that coding amino acids key genes were mutated in the process of evolution [20]. However, why this non-TIG type of CAMTA is universal needs further exploration.

Ankyrin repeats (ANKs), a domain involved in protein–protein interactions, contain 33 amino acids [36,37]. Most of the CAMTA3s (70.3%) have two ANK repeats in 37 plant species (Figure 2a). The repetitive motif IQXXXRGXXX of IQ motifs can bind with CaM and CaM-like proteins [38]. However, the number of IQ motifs in different plant species is uncertain. In Figure 2a, except for MtCAMTA3 and PpCAMTA3, all other CAMTA3 proteins were predicted to contain two or three IQ motifs. Rahman et al. (2016) reported that the ancestor of CAMTAs might contain two IQ motifs through analyzing 200 CAMTAs. CaMB, responsible for binding with Ca^2+^/CaM, is a motif that is unique to CAMTAs [20]. It exists in all plant CAMTA proteins except in *Caenorhabditis elegans* [4,19]. All CAMTA3 proteins were predicted to contain the CaMB domain (Figure 2a). Qiu et al. demonstrated that, when mutations lead to the loss of CaM-binding ability of CAMTA3, the effect on the plant’s defense against insect attack was similar to the effect of losing functionality of CAMTA3 [39].

Through multiple sequence alignment of CAMTA3, we found a highly conserved motif sequence: WXVX (2) [LVM] XKX (2) LRW [RLK] [RL] [KR] X (3) [FL] RX (Figure 2b). Bouche et al. (2002) and Yang et al. (2012) have suggested that CaMB of CAMTAs had the conserved motif sequence WXVX (2) LXKX (2) [LF] RWRX [KR] X (3) [FL] RX in *Arabidopsis* and tomato. A previous study in *Brassica napus* L. revealed that there was a conserved functional motif, namely, WXVX (2) L XKX (2) [LI] RWRXKX (3) [LF] [RKIV] X of CaMB [4]. The multiple alignment showed a conserved sequence for functional residues, i.e., WXVX (2) [LVI] XKX (2) [L] [R] [W] [R] X [KR] X (3) [FL] [R] X, in cotton CAMTAs [19]. Compared with the CaMB conserved sequence of CAMTAs previously reported, the 6th and 14th positions of the CaMB conserved sequence of CAMTA3 had M and LK residues, respectively, while the L and RL residues, respectively, occupied the 11th and 15th positions. These data demonstrated that the CaMB domain sequence was highly conserved in plant CAMTA3 proteins.

### 2.3. Gene Structure of CAMTA3

**Figure 3:**
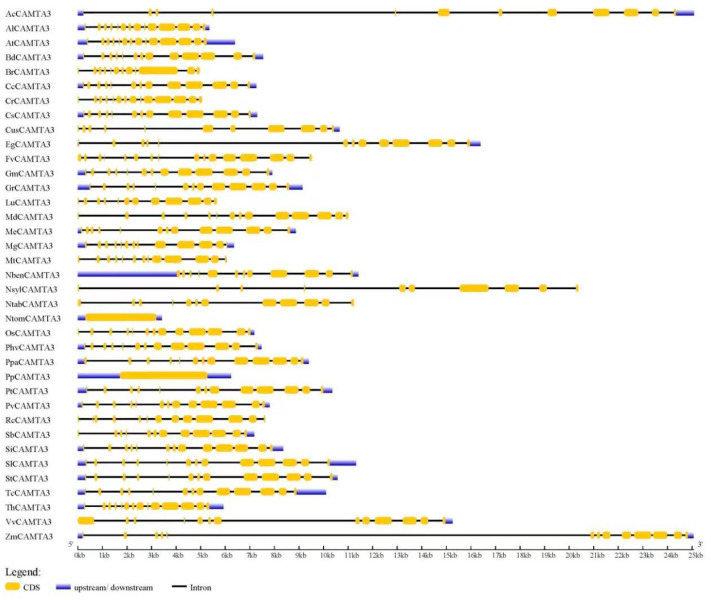
Gene structure of *CAMTA3/SR1* in different plant species. The untranslated regions (UTRs) are indicated by thick blue boxes; the exons are indicated by thick yellow boxes; the introns are indicated by black lines.

According to Figure 3, the *CAMTA3/SR1* gene structure was significantly different among 37 plant species. Among the 37 plant species, *AcCAMTA3*, *EgCAMTA3*, *NslyCAMTA3*, and *ZmCAMTA3* genes contained introns with an unusually large size (Figure 3; Table S1). This was also reported in *BnCAMTA3C2* and *BnCAMTA4A2* genes [28]. Further research is needed to explore whether those large introns have some special functions. *PpCAMTA3*, *NtomCAMTA3*, *NlsyCAMTA3*, and *FvCAMTA3* contained 0, 0, 9, and 15 introns, respectively. The numbers of introns of other plant CAMTA3 proteins ranged from 10 to 13 (Figure 3). This phenomenon occurs because during the evolution of the *CAMTA3/SR1* gene structure, there may be cases where genes are acquired or lost. A previous study also demonstrated that *BnCAMTAs* contained 11–14 introns, while *BnCAMTA4A2* contained 21 introns [28]. However, the CAMTAs gene structure includes 10–12 introns in many plant species, including *GmCAMTAs* [14], *PhvCAMTAs* [23], *MtCAMTAs* [15], *AtCAMTA3* [4], *ZmCAMTA3* [16], cotton*CAMTAs* [19], and *Nicotiana tabacum* [18]. Rahman et al. reported that the intron numbers of about 92.5% of 200 CAMTAs ranged from 10 to 13 [28]. Thus, the number of exons and introns in CAMTA3 are similar to those in plant CAMTAs; so the conservation in the gene structure of CAMTA3 is very important for plant species.

Figure 3 shows that the number of introns of SlCAMTA3 is 12, indicating that members of the CAMTA3 gene are conservative in the gene structure. However, studies on CAMTA3/SR1 in tomato as a transcription factor in plant growth and abiotic stress responses are limited. Thus, in the next step, we focused on the role of CAMTA3 in tomato.

### 2.4. Cis-Acting Element Analysis in the Promoters of the SlCAMTA3/SR1

**Figure 4:**
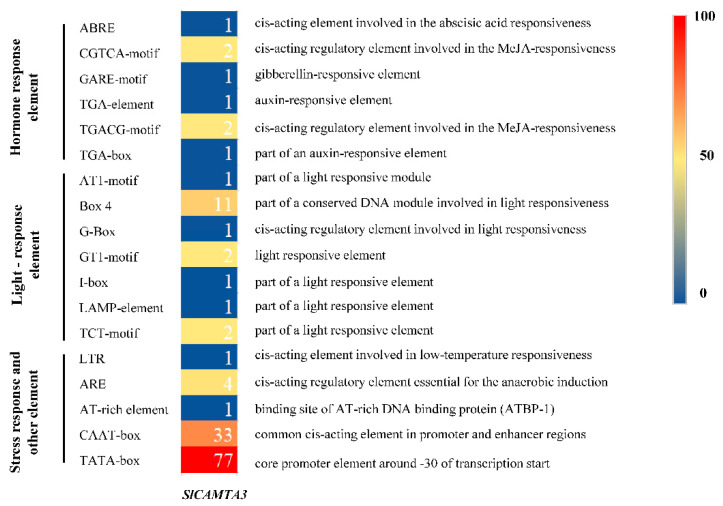
The distribution and function prediction of cis-acting elements in the upstream 2 kb regions of *SlCAMTA3/SR1* genes. The different colors and numbers of the grids indicate the number of different cis-acting regulatory elements in these *SlCAMTA3* genes, and their names are given on the left side of the diagram. The logos and annotations of functionally defined motifs are given on the right side.

Cis-acting elements in gene promoter regions are traditionally explored as binding sites for specific transcription factors that help regulate the initiation of gene transcription [40]. It plays a crucial role in the regulation of gene expression, especially for the regulation of gene expression in response to biotic and abiotic stress. To explore the possible response mechanisms of *SlCAMTA3/SR1* to various external stimuli, the cis-acting regulatory element of the promoter region of the *SlCAMTA3/SR1* gene was further investigated using a 2000 bp sequence upstream of the transcription start site. A total of 181 cis-acting elements were analyzed, and 17 were identified to be associated with plant hormones, light response, stress response, and tissue-specific expression (Figure 4). In terms of the different types of components, the most diverse are those related to the light-responsive component containing seven cis-acting elements, indicating that *SlCAMTA3/SR1* might play an important role in photo response. A previous study showed that CAMTA3 might be a negative regulator of auxin and may be associated with genes responsible for red light and high-light-level responses [5]. Notably, the tomato promoter EDS1 (Solyc06g071280) contains a CGCG box cis-element, which means that tomato contains a potential target of *SlCAMTA3/SR1* [24]. Combined with the phylogenetic relationship (Figure 1), we speculate that CAMTAs could play an important role in tomato growth. We also analyzed the hormone-related components, which include four cis-acting elements, the abscisic acid (ABA)–responsive element (ABRE), MeJA-responsive element (CGTCA-motif and TGACG-motif), auxin-responsive element (TGA-box and TGA-element), and gibberellin-responsive element (GARE-motif), finding that they may be sensitive to hormones. Additionally, there was only one stress-responsive component associated with low-temperature stress responsiveness (LTR), mainly related to low-temperature stress. Previous studies have shown that *AtCAMTA* genes are associated with cold stress [41], especially that *AtCAMTA1* and *AtCAMTA3* double mutants negatively affect freezing resistance [30]. More stress-related cis-elements have been reported to be located in the promoter regions of wheat CAMTA genes compared to other plant species [42], suggesting that wheat CAMTA genes may be more extensively involved in plant response to stress. Furthermore, other elements are also involved in the anaerobic induction, promoter and enhancer regions, and binding site of AT-rich DNA binding protein (ATBP-1). Therefore, more exploration is needed to reveal the detailed relationships between the different reactions of *SlCAMTA3/SR1* and the corresponding cis-acting elements.

### 2.5. Construction of Tomato CAMTA3 Protein Interaction Network Map

**Figure 5:**
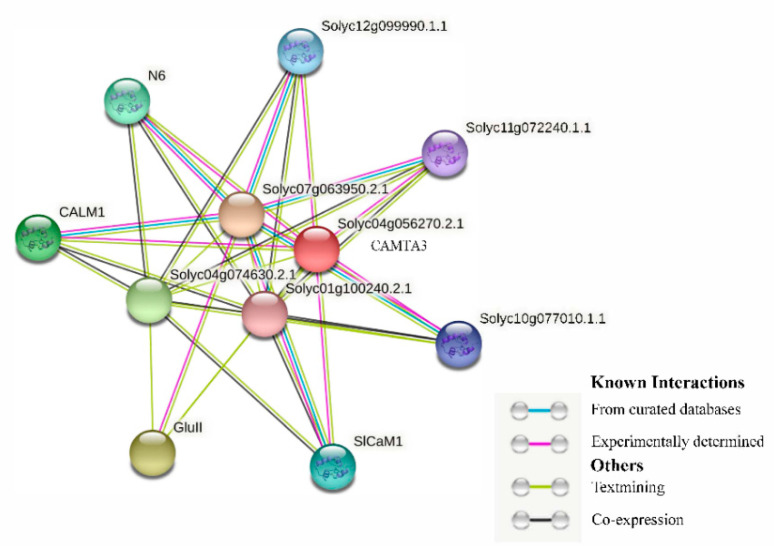
CAMTA3 protein clustering interaction network. Different colors represent various types of interactions. Network nodes represent proteins; empty nodes represent proteins with unknown 3D structures, while filled nodes represent proteins with known or predicted 3D structures. Edges represent protein–protein associations.

A study of the complex network of protein interactions led to the identification of a novel protein family with an important role in plant development, especially in plant meristem and leaf development [43]. The tomato CAMTA3 protein interaction network map was then predicted (confidence value = 0.5) (Figure 5). SlCAMTA3 protein is in the interaction network of known proteins in tomato, indicating a strong linkage between these proteins. The resulting highly connected network consists of more than 34 interactions amongst 10 proteins (Figure 5), and some of them, obviously interact with more than one protein and co-express. An earlier study showed that the 38 potential interactors of AtCAMTAs are related to Ca^2+^ signaling components, such as Ca^2+^/CaM-regulated protein kinases, Ca^2+^-dependent phospholipids, and CaM-binding proteins [20]. The present analysis similarly identified that the SlCAMTA3 protein mainly interactions with Ca^2+^ signaling components. In addition, different AtCAMTAs have redundant but not identical predicted interactors. Rahman et al. reported that AtCAMTA3 possesses 10 interactors, which are DNA-binding transcription factors, including SRS, CBP60G, CM2, ICE1, XLG2, RHL41/ZAT12, CBF1, CBF2, EDS1, and EDS16/ICS1 [20]. However, none of these proteins interacted with SlCAMTA3 in the present study. Thus, the regulatory pathway of the SlCAMTA3 protein is not the same as that of the AtCAMTA protein. The results indicate that the interactions of CAMTA proteins are distinct in different plant species.

### 2.6. Expression Analysis of SlCAMTA3/SR1

#### 2.6.1. The Expression under Different Treatments

**Figure 6:**
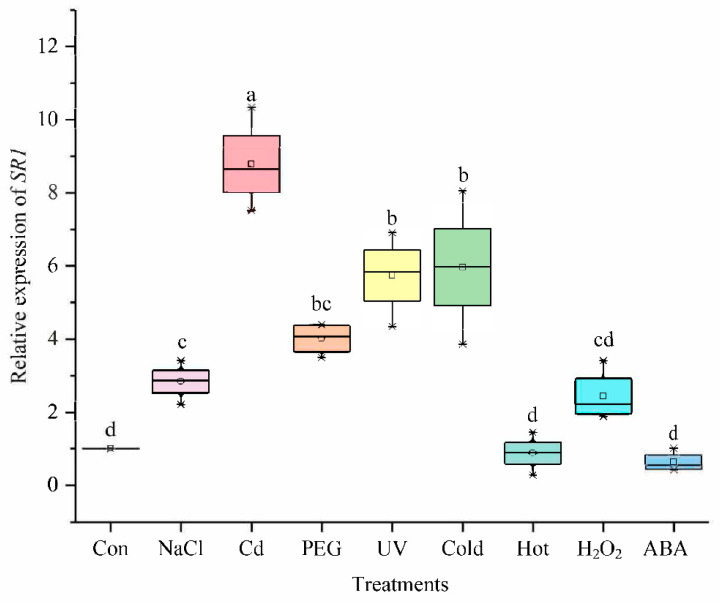
The expression levels of *SlCAMTA3/SR1* genes under different treatments. The central line for each box plot indicates the median. The top and bottom edges of the box indicate the 75th and 25th percentiles, respectively. The values with different letters are significantly different according to Duncan’s multiple tests (*p* < 0.05).

**Figure 7:**
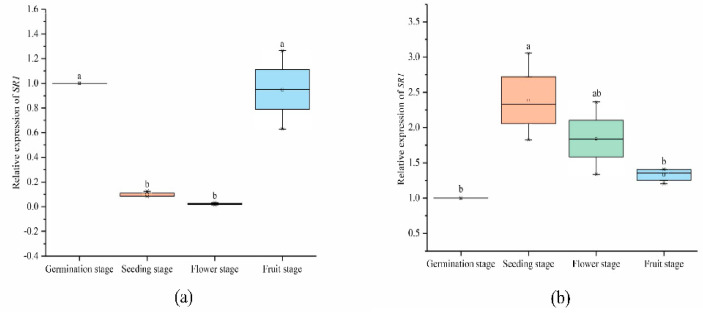
Expression levels of *SlCAMTA3/SR1* genes in the leaf (**a**) and root (**b**) at different stages. The central line for each box plot indicates the median. The top and bottom edges of the box indicate the 25th and 75th percentiles, respectively. The values with different letters are significantly different according to Duncan’s multiple tests (*p* < 0.05).

Several studies have shown that some members of the CAMTA family regulate different abiotic stress responses [7,44]. We confirmed the differential expression patterns of the *SlCAMTA3/SR1* gene under different treatments (Figure 6). The result showed that *SlCAMTA3/SR1* genes responded positively to various abiotic stresses, including NaCl, Cd, FEG, UV, and cold stresses. The expression of *SlCAMTA3/SR1* under Cd stress was significantly higher than that under the stress-free condition (the control) and other stresses, showing an eightfold increase over the control (Figure 6). It can be speculated that *SlCAMTA3/SR1* might respond to Cd toxicity. The previous study found that Al induced the expression of *AtCAMTA2*, which may alleviate heavy-metal toxicity in plants [45]. The specific mechanism of *CAMTA/SR* in Cd and other metal stresses has not been reported so far. Clearly, this mechanism should be further investigated in the future. Nevertheless, the expression of *SlCAMTA3/SR1* under heat stresses remained almost unchanged. *AtSR1* and *AtSR2* were shown to play an important role in regulating tolerance to low temperature [30,46], while few studies have been conducted to investigate its response to high-temperature stress. According to our result, *SlCAMTA3/SR1* may be insensitive to heat stress. Previous studies have confirmed that *CAMTA3* might play a major role in plant response to drought, low temperature, and salt stress in *Arabidopsis* and cucumbers [34,46,47], which is consistent with our results in tomato. The marked response of *SlCAMTA3/SR1* to the cold tolerance should be attributed to cold-stress-related cis-acting element (LTR) (Figure 4 and Figure 6). An interesting phenomenon is that *SlCAMTA3/SR1* does not have NaCl, Cd, PEG, and UV response cis-acting elements, but was upregulated by these stresses. Moreover, there was no significant difference in the *SlCAMTA3/SR1* gene expression among the control or the H_2_O_2_ and ABA treatments (Figure 7). *SlCAMTA3/SR1* contains an ABA response cis-acting element. This phenomenon was also observed in members of the soybean and cucumber CAMTA family [27,47], implying that the corresponding elements do not necessarily correspond to the occurrence of the reaction. The study could be enhanced if further analysis were done to explore the physiological relevance of this change over the treatment period, as well as its interaction with cis-acting elements and abiotic stress responses.

#### 2.6.2. Expression Analysis of *SlCAMTA3/SR1* Genes at Different Stages in the Reproductive Growth Period

Biological complexity is always based on the synergistic cooperation achieved by combinatorial interactions between the molecular components of the cell [48]. Previous studies have shown that CAMTA is involved in plant development in a Ca^2+^/CaM-driven modus [4,5,6,7,49]. In our study, the *SlCAMTA3/SR1* gene was differently expressed in the leaf and root at different stages (Figure 7). The expression of *SlCAMTA3/SR1* in the root and leaf at the germination stage was normalized to 1 (as a control). Most notably, the expression of *SlCAMTA3/SR1* was scarcely detected in tomato leaf at the seedling and flowering stages, whereas *SlCAMTA3/SR1* was not differently expressed at the germination and fruiting stages (Figure 7a). This phenomenon suggests that *SlCAMTA3/SR1* is involved in a series of metabolic pathways during early development and fruit ripening to maintain plant growth and development. However, the expression pattern of *SlCAMTA3/SR1* in the root was different from that in the leaf. *SlCAMTA3/SR1* was highly expressed in the root at the seedling stage, which was significantly higher than the expression of *SlCAMTA3/SR1* in the root at the germination and fruiting stages (Figure 7b). There was a slight increase but no significant expression difference compared with the control in the root at the flowering and fruiting stages. Plants continuously adjust their body plans to adapt to the local environment by forming new organs in various developmental stages [50]. We speculate that the high expression of *SlCAMTA3/SR1* in the root during the seedling stage was diminished in the leaf because of the plant’s need to absorb sufficient nutrients to maintain plant development and metabolic homeostasis strategies, and this gene should be involved and play a vital role in this process. The data reveal a complex tissue- and time-specific regulatory mechanism that may have specific roles in different growth periods and in various plant tissues. However, the period and intensity of action between members are different, and the specific functions need to be further verified.

#### 2.6.3. Expression Analysis of SlCAMTA3/SR1 Genes of Different Organs in the Reproductive Growth Period

**Figure 8:**
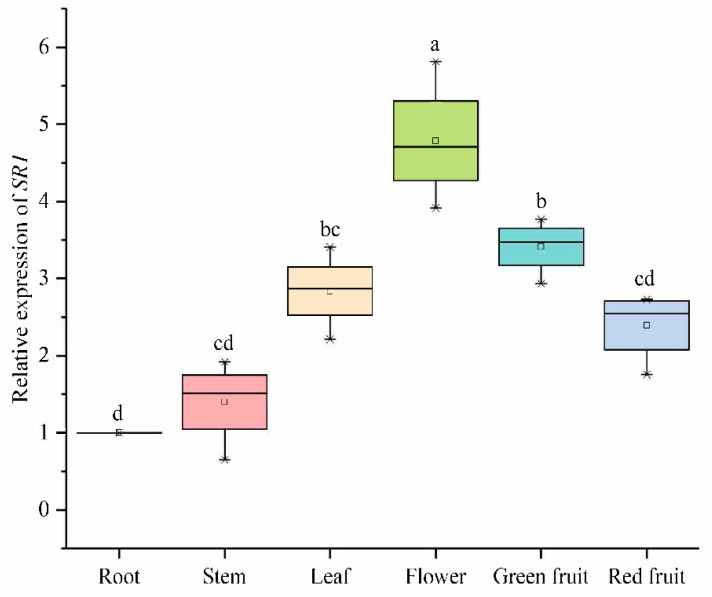
Expression levels of *SlCAMTA3/SR1* genes of different organs in the reproductive growth period. The central line for each box plot indicates the median. The top and bottom edges of the box indicate the 25th and 75th percentiles, respectively. The values with different letters are significantly different according to Duncan’s multiple tests (*p* < 0.05).

We analyzed the expression of *SlCAMTA3/SR1* in different tissue sites in tomato during the reproductive growth stages, which further clarified the important roles of *SlCAMTA3/SR1* during tomato growth and development (Figure 8). The expression of *SlCAMTA3/SR1* in the root at the reproductive stage was normalized to 1 (as a control). The expression level in the leaf, flower, and green fruit was significantly higher than that in the root. Chief among them, *SlCAMTA3/SR1* has the highest expression levels in the flowers, which was significantly higher than in that in the leaf and green fruit. Similarly, the expression investigation of *VvCAMTAs* in *V. vinifera* revealed that they are primarily involved in Ca^2+^ signal relay, with significantly higher expression in the bud, fruit, and inflorescence [13]. However, the expression levels of *SlCAMTA3/SR1* in the stem and red fruit are similar, with no significant difference compared to that in the root. In tomato (cv Rutgers), *SlSR1* had expression peaks that appeared at the mature green and red ripe stages [12]. Interestingly, *SlCAMTA3/SR1* was highly expressed in green fruit but diminished in red fruit during the reproductive growth stage (Figure 8). A previous study has shown that *SlSRs* operate as possible signal junctions for connection development and ethylene- and calcium-mediated signals during tomato fruit growth and maturation [12]. Thus, the differential expression patterns of *SlCAMTA3/SR1* were due to its differential regulation during fruit development and ripening. This is similar to the high expression levels of wheat *TaCAMTA1*-D and *TaCAMTA3*-D in reproductive ears and seedling buds, respectively [42]. Our findings suggest that *SlCAMTA3/SR1* may play important roles in the tomato leaf, flower, and green fruit during reproductive growth but has only minor effects on the stem and red fruit. Clearly, *SlCAMTA3/SR1* is expressed in all tomato tissues, and its expression levels change along with the life cycle and tissues. However, the period and intensity of action among organs are different, and this specific molecular mechanism needs further verification.

## 3. Materials and Methods

### 3.1. Identification of the CAMTA Family Members in Solanum lycopersicum

The *Solanum lycopersicum* genome sequence and protein sequence information file were downloaded from the National Center for Biotechnology Information (NCBI) (https://www.ncbi.nlm.nih.gov/ (accessed on 12 December 2021)) database. The phylogenetic tree was constructed using Clustalx program by the maximum-likelihood (ML) method with a bootstrap of 1000 in MEGA7 (Philadelphia, PA, USA) (http://www.megasoftware.net/ (accessed on 15 December 2021)). After the phylogenetic tree was built, we used Evolview (Wuhan, China) (https://evolgenius.info//evolview-v2/ (accessed on 20 January 2022)) to decorate it. The protein sequences that we needed were obtained from Phytozome (Goleta, CA, USA) (https://phytozome.jgi.doe.gov/ (accessed on 10 January 2022)), NCBI (http://www.ncbi.nlm.nih.gov/ (accessed on 15 January 2022)), PlantTFDB (Beijing, China; Peking University) (http://planttfdb.gao-lab.org/ (accessed on 2 February 2022)), and Gramene (the European Bioinformatics Institute: EBI) (http://ensembl.gramene.org/ (accessed on 5 February 2022)).

### 3.2. Gene Structure and Protein Conserved Domain Analysis

Phytozome (https://phytozome.jgi.doe.gov/), NCBI (http://www.ncbi.nlm.nih.gov/), PlantTFDB (http://planttfdb.gao-lab.org/ (accessed on 10 February 2022)), and Gramene (http://ensembl.gramene.org/ (accessed on 10 February 2022)) were used to search for protein sequences and gene sequences in plant species. The protein structure was analyzed online on the SMART database (Heidelberg, Germany) (http://smart.embl-heidelberg.de (accessed on 15 February 2022)). NLS motifs were researched using the Motif scan (Swiss) (https://myhits.isb-sib.ch/cgi-bin/motif_scan (accessed on 16 February 2022)) [19,20]. The Calmodulin Target Database (Canada) (http://calcium.uhnres.utoronto.ca/ctdb/ctdb/ (accessed on 20 February 2022)) and MEME Suite (Queensland, Australia) (http://meme-suite.org/ (accessed on 20 February 2022)) provided us with a CaM-binding site reference. The domain structure was painted using Domain Illustrator Software (Hefei, China) (http://dog.biocuckoo.org/ (accessed on 25 February 2022)) [51].

### 3.3. Cis-Acting Elements Analysis

Multiple sequence alignments and sequence logos of the CaMB domain were drawn using Geneious version 9.1 (Biomatters Ltd., Auckland, New Zealand) (http://www.geneious.com (accessed on 28 February 2022)). The corresponding consensus motifs were shown above with the sequence logo.

The DNA sequences of *SlCAMTA3* genes were obtained from the tomato genome sequence, and cis-acting elements in the promoter region were analyzed in the PlantCARE database for Flanders Institute for Biotechnology (VIB) (http://bioinformatics.psb.ugent.be/webtools/plantcare/html/ (accessed on 10 March 2022)).

### 3.4. Gene Structure of CAMTA3

These gene sequences were analyzed using the Gene Structure Display Server (Center for Bioinformatics (CBI), Peking University.) (GSDS, http://gsds.cbi.pku.edu.cn/index.php (accessed on 15 March 2022)) [52].

### 3.5. Construction of CAMTA Protein Interaction Network Map

Interaction network maps of tomato SlCAMTA3 proteins were constructed using the STRING (Hinxton, Cambridgeshire, UK) (http://STRINGdb.org/ (accessed on 15 March 2022)) online tool.

### 3.6. Plant Materials and Treatments

The seedlings of tomato *Lycopersicum esculentum* L. ‘Micro-Tom’ were used as plant material in this study. Seeds were surface sterilized with 1% NaClO before germination and transferred to 1/2 Hoagland solution for 7 days and then continued to be cultured in Hoagland solution for 21 days. Next, seedlings with identical size and growth were selected and treated. The light intensity of the growth chamber was 250 µmol photons m^−2^s^−1^, maintained at 26 ± 2 °C for 16 h during the day and 20 ± 2 °C for 8 h at night, with a relative humidity of 60% ± 5%.

The selected seedlings were transferred to Hoagland solution containing 150 mM NaCl, 200 μM Cd, 100 mM ABA, 10 % (*w/v*) (2.94 M) hydrogen peroxide (H_2_O_2_), or 20% (*w/v*) PEG6000. For cold and heat treatments, the seedlings in Hoagland nutrient solution were transferred to growth chambers under 4 or 40 °C. Some other selected seedlings were transferred to a growth chamber equipped with 253.7 nm UV-C radiation by a UV-C lamp (TUV PL-S 40 W/4P, Philips, Poland), and the growth conditions were the same as those for the control. Each treatment contained 3 biological replicates, with each replicate consisting of 12 seedlings. After 7 days of treatment, plants from each replicate were harvested separately, frozen in liquid nitrogen, and stored at −80 °C for subsequent experimental analysis.

### 3.7. RNA Extraction and Quantitative Real-Time PCR (qRT-PCR)

Total RNA was extracted from the samples using TRIzol reagent (Invitrogen, Carlsbad, CA, USA). The FastQuant First-Strand cDNA Synthesis Kit (Tiangen, Beijing, China) was used to synthesize cDNA according to the manufacturer’s protocol. qRT-PCR was performed using the LightCycler 480 Real-Time PCR System (Roche Applied Science) and SYBR^®^ Green Premix Pro Taq HS qPCR Kit and Roche LightCycler instrument. There were three biological replicates per treatment. The primers used for RT-PCR (F: 5′-ACG GTT TCT GAA AGG ACT GCT ACA C-3′ and R: 5′-GCA CCC TGA AGA CCT GAT GAA TAC G-3′) were designed using prime5 software. The tomato *Actin* gene was used to normalize relative expression levels. The 2-∆∆Ct calculation method was used to analyze the data. Statistical analyses were performed using the software SPSS 22.0 (SPPS Inc., Chicago, IL, USA). Statistical differences between measurements from different times or in different treatments were analyzed using Duncan’s multiple range test. Differences were considered significant at a probability level of *p* < 0.05.

## 4. Conclusions

Collectively, the CAMTA3 protein domains and genes of 37 plants were summarized using various online and offline bioinformatics tools. The CAMTA3 protein domain is similar in 37 plants, and the CaMB domain of CAMTA3 is highly conserved. However, the *CAMTA3* gene structure is significantly different in specific species. We suspect that this leads to some potential relationships between the various constituent domains of CAMTA3. Our predicted results prove that *SlCAMTA3/SR1* genes possess numerous cis-acting elements related to hormones, light response, and stress in the promoter regions. In addition, the SlCAMTA3 protein mainly interacts with Ca^2+^ signaling components, and the interactions of CAMTA proteins are distinct in different plant species. The expression patterns under hormonal and abiotic stresses indicate that *SlCAMTA3/SR1* responded positively to various abiotic stresses, especially Cd stress. Therefore, it can be speculated that *SlCAMTA3/SR1* might likely have a positive response to Cd stress, and this should be further investigated. The *SlCAMTA3/SR1* gene was differently expressed in the leaf and root at different stages. In addition, *SlCAMTA3/SR1* has the highest expression levels in flowers at the reproductive stage. The data reveal a complex tissue- and time-specific regulatory mechanism that may have specific roles in different growth periods and in various plant tissues. However, the period and intensity of action between members are different, and the specific functions need to be further verified. Our results provide basic reference into the phylogeny of CAMTAs in plants and the function of *SlCAMTA3/SR1* transcription factors in response to plant growth and abiotic stresses. In future, further in-depth studies are needed to elucidate the mechanisms of the differentially expressed *SlCAMTA3/SR1* genes in plant growth and development, as well as the regulatory mechanisms of signal transduction and stress-tolerance-related pathways. However, due to the possibility of functional redundancy or overlapping functions, molecular characterization of individual CAMTA genes remains a significant challenge for researchers. We can further combine experimental data with mathematical modeling to understand the information flow from Ca^2+^ signatures to CAMTA-regulated gene expression patterns.

## Data Availability

Data are contained within the article or Supplementary Materials.

## Supplementary Materials

The following supporting information can be downloaded at https://www.mdpi.com/article/10.3390/ijms23116264/s1.
